# The Process of Inflammation

**Published:** 1879-08

**Authors:** Kate C. Bushnell

**Affiliations:** Chicago


### ARTICLE IV.

### THE PROCESS OF INFLAMMATION. By KATE C. BUSHNELL, M.D., Chicago.

The phenomena of inflammation are most commonly studied by means of the microscope, in the tongue or mesentery of the frog, after injury by artificial means. They present themselves as follows:

The immediate result of the injury — if not too severe — is a spasmodic contraction of the blood-vessels. This is followed directly by a well-marked dilatation. The primary contraction may be absent, but the dilatation is never absent. It is, therefore, the first characteristic phenomenon observed, and is succeeded by an accumulation of white blood-corpuscles, or leucocytes, in the vicinity of the injury.

We next notice a slowing, then oscillation, and later, complete stasis of the blood current.

At this time the vessels become very irregular in outline and tortuous in direction. There is also a cloudy appearance of the tissues, and the parts are moist from the effusion of plasma about the seat of the injury. This association of phenomena, resulting from irritation, we call inflammation, and its striking physical sign is redness.

Examining the parts more closely, we find the tissues are studded with small, round corpuscles, resembling the leucocytes seen in the vessels.

Indeed, we might, by patient watching, see the leucocytes make their way through the walls of the capillaries into the surrounding structures. This passage of corpuscles through the capillary wall has been appropriately designated diapedesis.

After a time, varying from a few hours to several days, we find that the result of the inflicted injury is either resolution or suppuration.

In the former case, the blood current begins to move again, and the leucocytes are either carried away as leucocytes, or disintegrating, are absorbed and finally excreted from the system as waste matter.

In the case of suppuration, the corpuscles that have accumulated in the tissues surrounding the blood vessels rapidly proliferate and make for themselves a nidus by compressing and destroying adjacent structures.

And here we have an explanation of the swelling and pain that accompany inflammation. The swelling is caused by congestion and the abnormal growth of cells in the part. The pain results from pressure by the abnormal growth upon the nerve fibrils, and also from the disintegration of these fibrils in the general destruction which follows. Now this new growth, suspended in the plasmatic effusion, constitutes pus.

Nature generally forms a protecting membrane, by which this pus is separated from healthy tissue.

The resulting abscess may discharge spontaneously or be opened by artificial means; or the liquor of the pus may be absorbed, and the corpuscles, confined in their membrane, may disintegrate and form a cheesy deposit.

Should the abscess discharge its contents, the process of inflammation is completed by a growth of new tissues to fill the resulting cavity.

We have described the causes of three of the cardinal symptoms of inflammation: redness, swelling and pain. The cause of the fourth symptom — that of heat — is not so well understood. Owing to the fact that the temperature of an inflammatory focus is frequently higher than that of the blood which supplies it, the intense heat cannot be due simply to the determination of blood to the part, but must be produced locally. Probably it arises from the active change going on at that point.

We will now discuss the obscure points connected with inflammation, and see to what extent we can account for the phenomena observed. But in order to do this intelligently, it will be necessary to turn our attention for a short time to the histology of the structures involved — particularly the smaller blood-vessels. They are derived from the middle embryonic plate. Their inner layer is identical, as to structure, with the serous membranes, being covered with a peculiar cell called endothelium ; these cells or plates are polygonal in shape, and united at their circumferences. Between these plates — especially in the smaller arteries and veins — there are minute pores or stomata. They appear as if produced by a failure on the part of the endothelial plates to fit closely into each other.

The capillaries are entirely made up of this single layer, and are destitute of muscular fibers. During circulation, their caliber is regulated, to a considerable extent at least, by the amount of blood in the arterioles.

Around the capillaries, and also around the smaller arteries and veins, perivascular spaces have been recently demonstrated. They form small channels, which seem to be the beginning of the lymphatics. The caliber of these plasmatic channels is not large enough to admit the blood or lymph corpuscles. Besides this system, I believe there exists another set of channels, leading from the capillaries to the nuclei of all surrounding tissues.

The middle coat of the arteries and veins is a transverse muscular layer. Replaced by elastic tissue in the arteries of largest diameter, it is gradually developed as we approach the arterioles ; it then diminishes again, and in the smallest arterioles traces of it only can be found, as single, nucleated, spindle-shaped fibers wrapped around the endothelial layer. This muscular layer is entirely wanting in the capillary system; but as these vessels emerge into venules, it again appears, and is found highly developed throughout the venous system.

The blood-vessels are supplied by the vaso-motor portion of the sympathetic system. These fibers, which pass to the vessels, are non-medullated, and ganglia in connection with them are found in the muscular walls of the arteries and veins. These ganglia hold no unimportant place in the mechanism of circulation.

The distribution of the nerve-fibers is as follows: A nerve approaches a vein or artery, and divides into two branches, one of which crosses the vessel, and then the two accompany it on either side. These branches give off fibers which unite to form a plexus about the vessel, and from this plexus is given off another set of finer fibrillæ, which terminate in the vascular wall itself.

These nerves are both sensory and motor, and various functions have been abscribed to them.

We are quite sure of one function, at least: that is, the maintenance of vascular tonus. This condition is brought about in the following manner: the continual flow of blood through a vessel produces an impression which is conveyed to the neighboring ganglia by sensory fibers; thence it is reflected along motor nerves, causing a rigid condition of the walls of the vessel.

Applying our knowledge of the distribution and function of the nerves of the blood vessels, we can readily see how in the early stages of inflammation, interference by irritation might cause, in the first place, spasmodic contraction of the vessels followed by loss of vascular tonus resulting in dilatation or, at least, a flaccid condition of the vessels.

But we have stated that the early symptoms of inflammation are dilatation with acceleration of blood current. While the natural tendency of dilatation would be a slowing of the blood current. So that we must find a better cause than simple dilatation.

This dilatation and acceleration has been accounted for by the theory of collateral circulation. When some of the arterioles of a part are occluded or obliterated by injury, there will be an increase in pressure and blood-flow in the neighboring patent vessels. Doubtless such is the case when the injury is so great as to mangle or destroy the tissues, but very frequently in inflammation no such condition of things obtains. So that this explanation will not cover the whole case.

Goetz, Freusberg and others have been recently trying to demonstrate the existence of so-called vaso-dilator nerves. Should nerves of such function be proven to exist, the explanation will seem quite simple, for active dilatation of arteries would be quite liable to result in just the phenomena under discussion.

There is another very excellent theory advanced to explain these symptoms of active congestion. It is the theory of peristaltic action of the vessels, especially under irritation.

This kind of movement has been proven to exist in the blood vessels of some of the lower animals, and assists essentially in the propulsion of the blood. It has also been observed in the lymphatic of man.

If we adopt this theory, the whole process can be accounted for without difficulty.

First, the irritation from injury excites the arterioles to active peristaltic action, propelling the blood more rapidly through the vessels, and the disturbance in the vascular tonus allows the vessels to become greatly distended. At the same time, the plasmatic channels which connect the blood and lymph vessels dilate also, and there is a rush of the blood stream through the arterioles into the capillaries, widely dilating them, and thence the serum, particularly, passes through the stomata into the perivascular spaces and adjacent tissues, leaving the leucocytes collected in abundance in the vessels. There are other causes for the determination of leucocytes to these parts. In the normal circulation it can be readily observed that the leucocytes move slowly along, clinging to the wall of the vessels, and after injury this tendency is exaggerated, either by structural changes in the walls of the vessels, the corpuscles themselves, or perhaps both, so that they are easily hindered in their course. Beale states that the nuclei of the endothelial plates, under the abnormal stimulation, grow to such an extent as to project into the vascular tube and obstruct the leucocytes. The leucocytes thus block up the way, and the vessels become more and more distended and tortuous in direction.

The spasmodic peristaltic action soon becomes inefficient on account of the great mass of leucocytes clogging the vessels, and it is here that we recognize the to and fro movement of the current, until finally the effort is given up through exhaustion on the part of the nerves, and the blood ceases to move. After awhile the nerves may regain their vigor, and all at once the blood stream starts forward. It stops again, and this phenomenon is repeated several times. The circulation is again established, the leucocytes are gradually carried away, and the process ends in resolution. However, quite frequently the process is carried to a greater extreme.

At the time that exudation of plasma, and oscillation and stasis, are going on, very interesting changes may be observed in the conduct of leucocytes that will indicate that the injury is to result in suppuration. Arrested in their flow through the vessels, the leucocytes find time to take on new characteristics.

They multiply and exhibit the peculiar amœboid movements, and as the blood serum escapes through the widely dilated stomata into the plasmatic channels and surrounding tissues, the leucocytes also escape and are seen to crawl away from the vessel by amœboid movement. They are either crowded through by the impulse of the corpuscles of the passing blood stream, are carried through in the current of the escaping serum, or creep through by virtue of their own power of movement.

It is very evident that there is a mechanical cause for the exudation of serum during inflammation. But the effusion is said also to result from over-excitation of the trophic nerves. How these nerves act is still a mystery. But it is not a disputed question that certain nerves, cerebro-spinal in origin, when irritated, cause derangements in the amount of nutritive fluid supplied to a part. We have familiar illustrations of these disturbances in herpes at one extreme and decubitus or bed sores at the other.

The leucocytes, once escaped into the tissues and bathed in plasma, rapidly multiply, and we have pus as the result.

Do all pus corpuscles come from escaped leucocytes? Cohnheim thought they did; while Virchow contended that they were produced entirely from connective tissue corpuscles.

It is generally believed that they arise from a variety of sources, *i. e.*:

1. They are the leucocytes themselves and their progeny.
2. They are from the nuclei of the endothelial plates of the capillary vessels.
3. Lymph corpuscles also produce them.
4. Beale contends that there are minute particles of germinal matter in the serum of the blood, that grow to form them.
5. They are from the connective tissue corpuscles; and
6. The nuclei of all surrounding tissues, bone, muscle, etc., except perhaps nerve nuclei, give rise to them.

We have described the plasmatic channels that lead from the capillaries to the nuclei of all surrounding tissues. We can easily see how favorable this arrangement is to the production of pus from these sources, when nutritive fluid is carried to them so directly, and, during inflammation, in such abundance.

